# Seroprevalence and factors associated with SARS-CoV-2 infection among healthcare workers: cross-sectional study

**DOI:** 10.1186/s12879-023-08760-5

**Published:** 2023-11-06

**Authors:** Watheq Thabet Taher, Amen A. Bawazir, Talal A. Sallam, Khaled Alsurimi

**Affiliations:** 1 Faculty of Medicine and Health Sciences, The University of Aden, Aden, Republic of Yemen; 2 College of Medicine, AlMaarefa University, Diriyah, Saudi Arabia; 3 Faculty of Medicine, Al-Baha University, Al Bahah, Kingdom of Saudi Arabia; 4 University of Doha for Science and Technology, Doha, Qatar

**Keywords:** Seroprevalence, COVID-19, SARS-CoV-2, Healthcare workers, COVID-19 vaccines

## Abstract

**Background:**

Healthcare workers (HCWs) are at a higher risk of contracting COVID-19 due to their close contact with infected patients. However, the true burden of COVID-19 among HCWs in Yemen is unknown due to the inadequate availability of healthcare and the subclinical nature of the disease. This study aims to estimate the seroprevalence of SARS-CoV-2 infection among HCWs in two Yemeni governorates and identify associated factors using a cross-sectional design.

**Method:**

A total of 404 HCWs were surveyed from June 2022 to September 2022 in Lahj and AL-Dhalea hospitals. A self-administered questionnaire collected demographic data, COVID-19 infection history, and vaccination status. A total of 404 human sera were tested using a specific electrochemiluminescence immunoassay assay. Association analysis was conducted to identify associations between antibody prevalence and demographic and vaccine-related variables.

**Result:**

The median age of the HCWs was 31 (Range 20–64) years, with 65.0% being male and 35.0% female. Of all HCWs, 94% were SARS-CoV-2 seropositive and 77.0% had no confirmed test of COVID-19-related symptoms. There was no significant association between seropositivity and demographic factors such as age, gender, occupation, or COVID-19 vaccination (*P* > 0.05).

**Conclusion:**

The seroprevalence of SARS-CoV-2 was high among HCWs in Yemen, primarily due to natural infection rather than vaccination. Compliance with infection prevention and control measures did not significantly affect seropositivity. This study highlights the need for improved healthcare systems and resources to reduce the burden of COVID-19 and promote infection prevention and control (IPC) measures among HCWs in Yemen.

## Introduction

The coronavirus diseases-19 (COVID-19) pandemic has rapidly spread across the world, with devastating effects on public health, economies, and societies. With its five consecutive waves of infection, it resulted in over 693 million confirmed cases and 6.9 million deaths as of August 14, 2023 [1]. In response, countries implemented several measures, including lockdowns, social distancing, and travel restrictions, while awaiting the development of vaccines, which have now been administered to millions of people worldwide [2].

Based on the guidelines of the World Health Organization (WHO), several hospitals commenced their strict implementation of strategies to protect their HCWs. These included providing adequate personal protective equipment (PPE), implementing infection prevention and control (IPC) measures, and regular screening of staff [3, 4]. Despite measures to protect healthcare workers, the burden of COVID-19 infections among them remained high [5, 6]. Therefore, the protection of HCWs requires both the application of the hierarchy of controls for IPC in healthcare settings and public health measures aimed at reducing COVID-19 transmission [7, 8]. HCWs face increased patient volumes and longer shifts, leading to exhaustion, burnout, and physical and mental stress, which puts them at risk of non-compliance with recommended infection prevention and control measures. Serological assays for SARS-CoV-2 have shown variable seroprevalence among HCWs, reflecting differences in time and region [9]. The findings of this assay based on detecting antibodies from prior exposure to the virus whether due to asymptomatic infection or an overt COVID-19.

Over the first three years of the pandemic variable seroprevalence among HCWs have been reported worldwide with different epidemiological models related to person-place and time. In early studies of the pandemic mainly in the year 2020, a COVID-19 seroprevalence of 14.8% was reported among HCWs in Saudi Arabia [10], 19% in Turkey [11], 25.6% in Egypt [12], 27% in New York City [13], 42.7% in Poland [14], 45% in Nigeria, [15], and 48% in Ethiopian [16]. As of late 2021, higher seroprevalence was reported among HCWs as it reached 89.3% in Hong Kong [17], and 94.5% in Delhi India [18]. This progressive increase of seroprevalence with time suggests an expansion of seropositivity rates although the extent, effectiveness, and role of this seropositivity in immunity to infection remained to be resolved. However, the available evidence is that the vaccine induced immunity seems to have played a part in at least reducing the disease severity. Uncertainty and underestimation of the true number of cases in many countries may be due to insufficient testing of all suspected cases [19]. Given all this, the HCW working in the frontline of healthcare settings would be highly exposed to COVID-19 during the different waves of the pandemic.

Yemen reported its first COVID-19 case on April 28, 2020, with over 11,000 infections and 2,159 deaths reported to date [8, 20]. Despite the low global case fatality (1.0%), COVID-19 case fatality rate in Yemen reached as high as is 22.6% [20]. The high case fatality rate in Yemen suggests that the actual number of cases and deaths may be much higher than official figures, and underreporting remains a concern due to the multitude of challenges Yemen still facing, including the political instability, conflict, and humanitarian crisis an well as lack of developing reliable epidemiological surveillance and reporting system [20, 21].

In mid-2020 COVID-19 cases in Yemen were estimated to have reached 1 million and this figure was predicted to reach 11 million with 85,000 deaths at the end of the year 2020 if people do not make serious behavioural changes, and if authorities do not introduce more mitigation to control the infection [22, 23]. However, according to the WHO reports from April 2020 to 13 January 2023, there have been 11,945 confirmed cases of COVID-19 with 2,159 deaths [23]. These figures indicate underreporting and thus uncertainty about the actual epidemic status of COVID-19 in the country. The political conflict left Yemen with many challenges to develop reliable epidemiological reports on the actual COVID-19 status due to inadequacy of epidemiological surveillance, lack of laboratory capacity and absence of community cooperation with the epidemiological surveillance teams. However, a study conducted among HCWs in Yemen during the COVID-19 pandemic illustrated that healthcare system capability and general preparedness to face COVID-19 was rated as very poor or poor by the majority of HCWs who participated in the study [24]. This is consistent with international reports, which show that Yemen's healthcare system is fragile and has limited capacity to cope with public health emergencies [25].

The situation among frontline teams in Yemen has not been fully assessed as yet. Thus, this study aims to investigate the seroprevalence of SARS-CoV-2 infection among health care workers (HCWs) in hospitals of two Yemeni governorates and identify associated factors.

## Methods

### Study design and setting, and study population

**Fig. 1:**
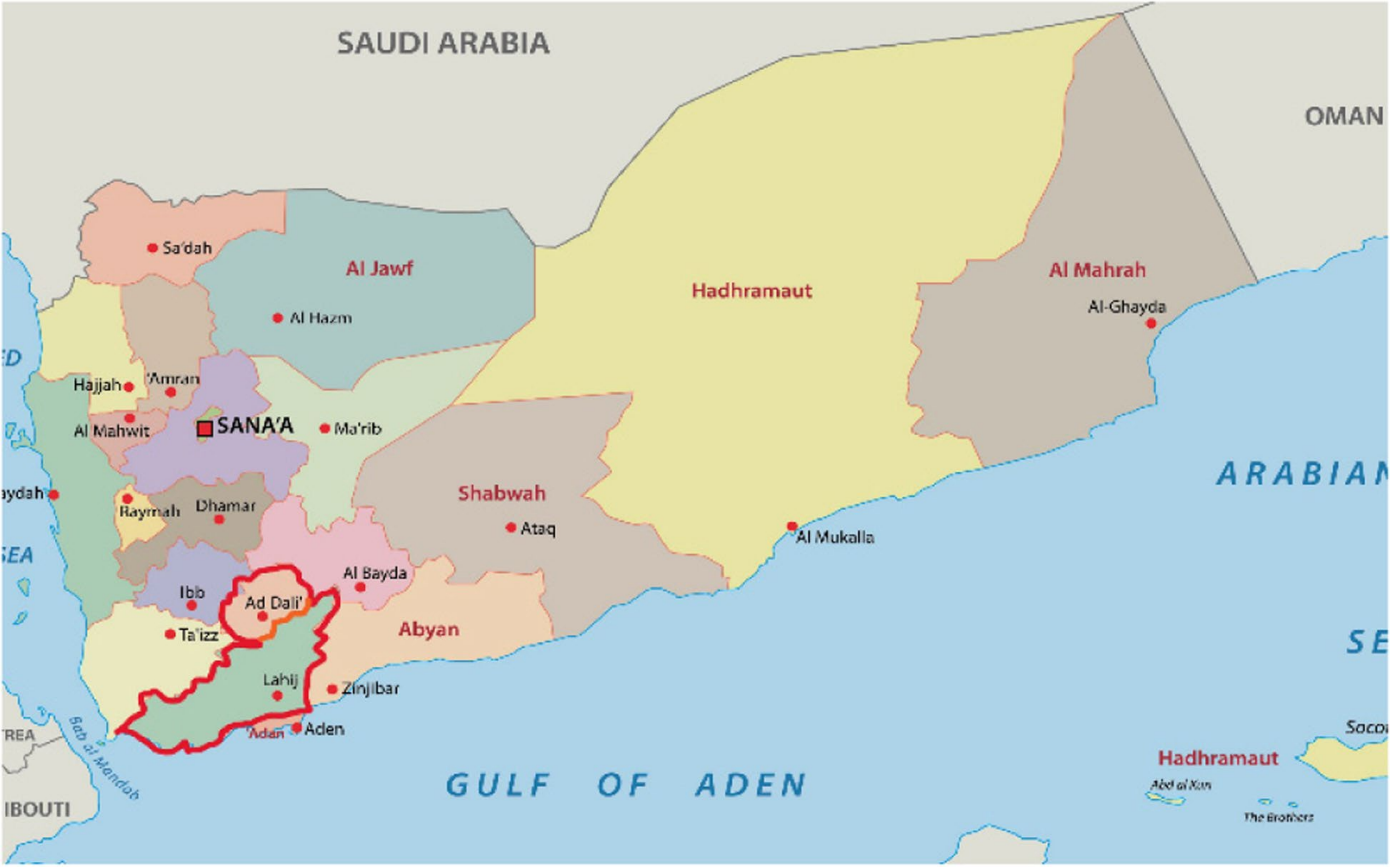
Map of Yemen identifying the areas of the Lahej and Aldalea Governorate

This is a cross-sectional study conducted in the hospitals located of Lahj and AL-Dhalea governorates in the southern part of Yemen in the period from June 2022 to September 2022 (Map of Yemen identifying the study areas). Lahj governorate, with a population of nearly 900,000 people, is located northwest of major southern city of Aden and is divided into 15 administrative districts with the city of Al-Hawtah as its capital. Al-Dhalea governorate, with a population of almost 500,000 people, is located in the southern-central part of Yemen with a population of nearly 500,000 people and encompasses nine administrative districts, with Al-Dhalea as its capital, [26] as seen in Fig. 1 [27].

The study population included a range of HCWs, such as doctors, nurses, x-ray physicians, dentists, laboratory personnel, pharmacists, respiratory therapists, and nutritionists, in addition to auxiliary HCWs such as clerks, housekeeping staff, laundry personnel, and social workers. Those who didn't agree to participate in the study and did not fill in the questionnaire were excluded with around 12 out of the total 416 (2.9%). However, participants were gathered into four categories according to the importance of the participants and the level of contact with patients complaining of COVID-19 infection such as physicians, nurses, allied health workers who involved in giving healthcare services distinct from medicine or nursing [28], and finally those categorized as support services, as people responsible for providing and maintaining a sanitary and therapeutic environment in which health care can be appropriately delivered to individuals [29]. Therefore, allied health workers included those working in the x-ray department, laboratory, and pharmacy accounting around 92 workers (22.8%). While those working in supporting services section such as cleaning and laundry personnel, maintenance, admission/reception clerks, patient transporters social workers, and housekeeping with a total of 58 participants (14.4%).

To recruit the participants, the research team obtained permission from the hospital administration in each governorate to conduct the study [30], as well as the work was approved by the Ethics Research Committee (ERC) of the Faculty of Medicine and Health Sciences, University of Aden (REC-#119–2022). An informed consent was given to the participants and the study team provided a brief overview of the study objectives to potential participants and requested their voluntary participation. Those who agreed to participate were asked to complete a self-administered questionnaire and provide a blood sample for serological testing. The questionnaire collected information on demographic characteristics, work-related factors, COVID-19-related symptoms, and exposure to COVID-19 patients or suspected cases.

### Sampling and sampling technique

The prevalence of anti-SARS-CoV-2 antibodies among healthcare workers in Yemen is not well understood, so a large sample size was needed to estimate it accurately. Based on an assumed prevalence rate of 50%, a 95% confidence interval, and a 5% margin of error, a sample size of 384 was calculated using Daniel’s equation [31]. To account for possible missing data or participant dropouts, an additional 5% of the sample size was added, resulting in a final sample size of 404 HCWs.

To ensure a representative sample, we used the Probability Proportional to Size (PPS) sampling technique to enroll participants from both Lahj and Al-Dhalea governorates proportionally. Of the HCWs who presented in the hospitals during the sampling period, 267 (66%) from Lahj and 137 (34%) from Al-Dhalea were enrolled in the sample and a convenience sampling method was used to complete the required sample from each hospital and governorate.

### Data collection

The study used a self-reported questionnaire based on the WHO protocol for COVID-19 infection among HCWs in a healthcare setting [4]. The HCWs completed the questionnaire in front of the investigator for any assistance needed by the participant and before the collection of the blood sample (8–12 min average). The questionnaire comprised of five domains, including demographic data, data related to exposure to COVID-19, COVID-19 symptoms and PCR confirmed or suspected infection, comorbidities, IPC measures, and COVID-19 vaccination. The first domain encompassed the demographic data including age, sex, healthcare setting, work experience, and occupation category. The second domain included data related to exposures to COVID-19 and these consisted of the frequency of exposure, time of occurrence, and the setting as a source of infection. The third domain comprised COVID-19 symptoms and PCR confirmed or suspected infection. The fourth domain consisted of data of comorbidities encompassing asthma, heart disease, hypertension, kidney disease, *diabetes mellitus*, and immune deficiency. The fifth domain encompassed data on IPC measures which included follow IPC standard precautions, following 5 recommended moments, use alcohol-based hand rub or soap, wear PPE with the COVID-19 patient, PPE available in sufficient quantity, and attended IPC training. In addition, this domain includes variables related to COVID-19 vaccination which included receiving of vaccine, acceptance, or hesitancy to receive the vaccine.

The questionnaire was originally in English and was translated into Arabic for ease of understanding and back to English to secure the consistency of the questions.

#### Pre-testing

Reliability test was undertaken among 30 HCWs in analogy to the site of the study to ensured that the questionnaire was easy to use and acceptable by the interviewees. Reliability also was considered by reaching a Cronbach alpha of not less than 0.73 of the completed questionnaires which indicates that the overall response values for each participant across a set of questions are consistent.

### Laboratory investigation

A volume of five ml venepuncture blood sample was drawn from each participant via the venipuncture technique with universal precautions conducted by the concerned technician. Blood samples were collected in EDTA tubes and stored at 4 °C and transferred on the same day to the hospital laboratory for analysis. The samples were labelled with the department name, name of the participant, date, and identification number [4]. Sera were tested for anti-SARS-CoV-2 using Elecsys Anti-SARS-CoV-2 Qualitative assay for use on the Cobas, Roche Diagnostics GmbH, electrochemiluminescence immunoassay (ECLIA) according to the instructions of the manufacturer. The assay has a sensitivity of 99.81% (CI 95%: 99.6–99.9%) and a specificity of 99.5% (CI 95%: 98.63–99.85%) and is certified by WHO [32, 33]. The assay provides a qualitative detection of all antibody classes (including IgG) to SARS-CoV-2 in human serum and plasma and is intended for use as an aid in identifying individuals with an adaptive immune response to SARS-CoV-2, for recent or prior infection. It can detect the presence of anti-SARS-CoV-2 antibodies in serum within days to weeks following acute infection [34, 35]. Sera with a Cut off Index (COI; signal sample/cut-off, COI) ≥ 1.0 were considered positive, those with a COI < 1.0 were considered negative.

### Data management and statistical analysis

The data was coded and entered into SPSS version 23 for analysis. Descriptive statistics, including mean and standard deviation, were used to summarize continuous variables, while absolute and relative frequencies were used for categorical variables. The Chi-square test was used to examine the association between the dependent and the independents variables. A *P*- of < 0.05 was considered statistically significant.

## Results

**Fig. 2:**
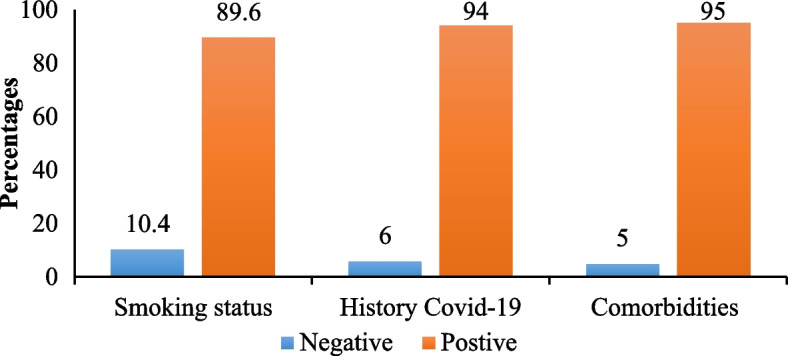
Seroprevalence of anti-SARS and risk factors

Of 404 HCW, 264 (65.3%) were male whereas 140 (34.7%) were female, the median age was 31 years (Range 20–64) years. Nurses constituted the highest proportion (51.2%) while physicians comprised the lowest proportion (11.6%). The overall seroprevalence of SARS-CoV-2 was 94.3%; 95%CI = (92.1–96.5%). Seroprevalence was similarly high and did not significantly differ (*P* > 0.05) between; genders, age groups, work experience, different occupation categories (Table 1). No significant association (*P* > 0.05) between the seroprevalence and the reported risk factors such as smoking, comorbidities including, cardiovascular diseases, history of diabetes mellitus, or renal diseases with a reported history of COVID-19 (Fig. 2). Further, seroprevalence was similarly high and did not differ significantly (*P* > 0.05) between different governorates, districts, hospitals (Table 2), those who did or did not adhere to IPC measures recommended by the WHO and those who attended and who did not attend IPC training course (Table 3). There was a convergently high seroprevalence (≥ 90.0%) which did not differ significantly (*P* > 0.05) among HCW who reported or denied history of COVID-19, breathing difficulty, cough, anosmia or loss of taste or fever/ chills, (Table 4), positive or negative history of close contact with confirmed COVID- 19 cases or with overt COVID-19 (Table 5). Among those who reported previous history of COVID-19 (*n* = 112) only 22 i.e., 5.4% of total HCW reported that they were tested by PCR and were all positive. Among all HCWs, 12.0% received one vaccine dose, 11.5% received two doses’, 76.5% have not received any vaccine. Of all HCWs 95 (23.5%) were vaccinated, 66 (70%) with AstraZeneca vaccine and 29 (30%) with Jenson & Jenson/Osinovac. More than half of the HCWs (58.9%) reported lack of desire to receive COVID-19 vaccine, while 9.9% will think too carefully before they take it. Equally high seroprevalence (> 93.0%) was not significantly (*P* > 0.05) associated with vaccine uptake, vaccine type or number of vaccine doses (Table 5).

**Table 1:** Association between demographic characteristics of the participants and the SARS-CoV-2 seropositivity (*n* = 404)

Variables	Categories	Total	Positive	Negative	*P* value
		**No. (%)**	**No. (%)**	**No. (%)**	
Sex	Male	264 (65.3)	246 (93.2)	18 (6.8)	0.180
	Female	140 (34.7)	135 (96.5)	5 (3.5)	
Age	≤ 25 years	52 (12.9)	49 (98.0)	3 (2.0)	0.724
	25–44 years	294 (72.8)	276 (93.8)	18 (6.2)	
	≥ 45 years	58 (14.4)	56 (96.6)	2 (3.4)	
Duration in service	1 year	12 (3.0)	12 (100.0)	0 (0.0)	0.687
	2 to 5 years	190 (47.0)	179 (94.2)	11 (5.8)	
	> 5 years	202 (50.0)	190 (94.0)	12 (6.0)	
Occupational category	Physicians	47 (11.6)	43 (91.5)	4 (8.5)	0.648
	Nurses	207 (51.2)	195 (94.2)	12 (5.8)	
	Allied health workers	92 (22.8)	87 (94.2)	5 (5.4)	
	Supporting services	58 (14.4)	56 (96.6)	2 (3.4)

**Table 2:** Seroprevalence rate among healthcare workers in the study settings and hospitals

Variables	Categories	Seropositive	Seronegative	*P* value
		**No. (%)**	**No. (%)**	
Governorate	Lahj	256 (95.8)	11 (4.1)	0.057
	Al Dhalea	125 (91.2)	12 (8.8)	
District Name	Radfan	33 (100.0)	0 (0.0)	0.207
	AL-Hoota	98 (96.0)	4 (4.0)	
	AL-Shaib	23 (96.0)	1 (4.0)	
	Taban	69 (96.0)	3 (4.0)	
	Yefea	20 (91.0)	2 (9.0)	
	AL-Dhalea	58 (90.6)	4 (9.4)	
	Towr albahah	18 (90.0)	2 (10.0)	
	AL-Azariq	28 (87.5)	4 (12.5)	
	Gehaaf	15 (83.0)	3 (17.0)	
Hospital	AL-Had	18 (100.0)	0 (0.0)	0.347
	AL-Wahat	15 (100.0)	0 (0.0)	
	Radfan	23 (100.0)	0 (0.0)	
	Ibn Kholdon	162 (95.8)	7 (4.2)	
	AL-Shaib	31 (94.0)	2 (6.0)	
	AL-Naser	82 (91.0)	8 (9.0)	
	14 October	20 (91.0)	2 (9.0)	
	Towr albahah	18 (90.0)	2 (10.0)	
	AL-Salam	12 (85.7)	2 (14.3)

**Table 3:** Adherence to infection prevention and control and personal protective measures in association with the seropositive tests

**Variables**	**Category**	**Total**	**Seropositive**	**Seronegative**	***P*-value**
		**No. (%**)	**No. (%**)	**No. (%**)	
Follow IPC standard precautions	Yes	314 (77.7)	298 (95.0)	16 (5.0)	0.333
	No	90 (22.3)	83 (92.0)	7 (8.0)	
Following 5 recommended moments	Yes	273 (67.6)	260 (95.0)	13 (5.0)	0.244
	No	131 (32.4)	121 (92.0)	10 (8.0)	
Use alcohol-based hand rub or soap	Yes	341 (84.4)	322 (94.0)	19 (6.0)	0.807
	No	63 (15.6)	59 (93.5)	4 (6.5)	
Wear PPE with the COVID-19 patient	Yes	289 (71.5)	275 (95.0)	14 (5.0)	0.243
	No	115 (28.5)	106 (92.0)	9 (8.0)	
PPE available in sufficient quantity	Yes	72 (17.8)	71 (18.6)	1 (4.3)	0.082
	No	332 (82.2)	310 (81.4)	22 (95.7)	
Attended IPC Training	Yes	154 (38.1)	148 (96.0)	6 (4.0)	0.221
	No	250 (61.9)	233 (93.0)	17 (7.0)	
The overall level of adherence to IPC	Poor	210 (52.0)	194 (50.9)	16 (69.6)	0.082
	Good	194 (48.0)	187 (49.1)	7 (30.4)

**Table 4:** Association between symptoms of Covid-19 infection and prevalence of positive tests

Variables	Category	Total	Seropositive	Seronegative	*P*. value
		**No. (%)**	**No. (%)**	**No. (%)**	
Breathing difficulty	YES	55 (14)	52 (94.5)	3 (5.5)	0.535
	No/NA	349 (86)	329 (94.3)	20 (5.7)	
Cough	YES	93 (23)	88 (94.6)	5 (5.4)	0.881
	No/NA	311 (77)	293 (94.2)	18 (5.8)	
Anosmia or loss of or taste	YES	104 (26)	97 (93.3)	7 (6.7)	0.596
	No/NA	300 (74)	284 (94.7)	16 (5.3)	
Fever/chills	YES	93 (23)	80 (93)	6 (7)	0.477
	No/NA	311 (77)	301 (94.7)	17 (5.3)	
Fatigue and weakness	YES	88 (22)	80 (93)	6 (7)	0.536
	No/NA	316 (88)	301 (94.7)	17 (5.3)	
Headache	YES	86 (21)	81 (92)	7 (8)	0.301
	No/NA	318 (79)	300 (94)	16 (5.1)	
Back and joint pain	YES	98 (24)	91 (92.9)	7 (7.1)	0.973
	No/NA	306 (76)	290 (94.8)	16 (5.2)	
Runny nose	YES	60 (15)	54 (90)	6 (10)	0.119
	No/NA	344 (75)	327 (95.1)	17 (4.9)	
Diarrhoea or vomiting	YES	33 (8)	31 (93.9)	2 (6.1)	0.881
	No/NA	371 (92)	350 (94.3)	21 (5.7)	

^a^Not applicable

**Table 5:** Association between COVID-19 infection and vaccine status with seropositive tests

Variables	Category	Total	Seropositive	Seronegative	*P*. Value
		No (%)	No (%)	No (%)	
**Infection status**					
contact with a + ve case	YES	196 (48.5)	185 (94.5)	11 (5.5)	0.964
	No	208 (51.5)	196 (94.0)	12 (6.0)	
History of covid-19	YES	112 (28.0)	105 (94.0)	7 (6.0)	0.765
	No	292 (72.0)	276 (94.5)	16 (5.5)	
**Vaccination status**					
Received vaccine?	YES	95 (23.5)	91(95.8)	4 (4.2)	0.476
	No	309 (76.5)	290 (93.9)	19 (6.1)	
Type of vaccine?	AstraZeneca	66 (16.0)	62 (93.9)	4 (6.1)	0.569
	Jenson & Jenson/Osinovac	29 (7.5)	29 (100.0)	0 (0.0)	
	Not received vaccine	309 (76.5)	290 (93.9)	19 (6.1)	
How many doses?	One dose	48 (12.0)	45 (93.8)	3 (6.3)	0.533
	Two doses	47 (11.5)	46 (97.9)	1 (2.1)	
	Not applicable	309 (76.5)	290 (93.9)	19 (6.1)	
Vaccine acceptance	YES	36 (8.9)	36 (9.4)	0 (0.0)	0.334
	NO	238 (58.9)	221 (58.0)	17 (73.9)	
	I will think carefully	40 (9.9)	38 (10.0)	2 (8.7)	
	N/A	90 (22.3)	86 (22.6)	4 (17.4)	

^a^Not applicable

## Discussion

Our study found a significantly high seroprevalence rate of SARS-CoV-2 (94.3%) among HCWs in Yemen between June 2022 and the end of September 2022, after the peak of the COVID-19 epidemic in the country [36]. This is a significant increase compared to a previous study conducted during the first peak of the pandemic in the southern city of Aden between November and December 2020, which reported a seroprevalence rate of 27.5% [21]. Earlier during the pandemic, another study among general population which was conducted between June 2020 through January 2021 in the capital Sana’a, Yemen, showed 51.4% seroprevalence among COVID-19 suspected patients [37]. The high seroprevalence among HCW in this study reflects the reality that HCWs are at a high-risk of acquiring SARS-CoV-2 infection given their direct role in patient care [38]. This is in line with reports from Saudi Arabia where seroprevalence among HCW in December 2020 was 10-folds higher (26.5%) than that among general population (2.36%) in May 2020 [39, 40]. Another study from South Africa (Gauteng Province), illustrated a period prevalence of SARS-CoV-2 infections ranged from 6.1% to 15.4% for the period 1 June—31 August 2020 [41].

Due to the political status in Yemen, epidemiological containment measures were inadequately implemented in the country. In fact, this study reported a lack of PPE in around 80.0% of HCWs despite reporting a high level of compliance with other IPC measures. The lack of PPE made compliance with other IPC measures ineffective, and this was translated into a seroprevalence that is as high as that among those reported non-compliance. This favours to some extent the inference of double exposure of HCW which occurred within and outside the health care setting in the community where poor containment measures were practiced. All these factors and others such as poor financial resources likely accelerated the spread of the virus among the HCWs and ultimately increased the pressure on the healthcare settings that lacked access to appropriate PPE and practiced limited compliance to IPC measures and ultimately intensely exposed HCWs to infection [24].

The scenario of exposure outside the health care setting is more likely to have occurred as the seroprevalence among auxiliary HCW such as clerks, housekeeping staff, laundry personnel, and social workers who literally has less contact with patients was as high as 96%.

The present study did not find a significant difference in seropositivity between HCWs who received the COVID-19 vaccine and those who did not. This is, realistically, due to the fact that the assay used in this study only detects anti-nucleocapsid antibodies [32, 33] while the existing COVID-19 vaccines only use the spike antigen. It is worth noting that the vaccination coverage with COVID-19 vaccine in Yemen is extremely low, as of 2 January 2023, a total of 1,242,982 vaccine doses have been administered in a country of 32 million population. The high seropositivity in our present study is likely due to natural infection rather than vaccination. It is reasonable to expect that the HCW could have produced anti-spike antibodies in even a higher proportion, as other studies reported that higher non-severe COVID-19 patients seroconverted to anti-spike antibodies than anti-nucleocapsid antibodies which declined more rapidly compared to anti-spike antibodies [38, 42].

Therefore, given the nature of the assay used in this study as it only detects anti-nucleocapsid antibodies [32, 33] this assay may have underestimated the actual seroprevalence and thus a higher proportion of the HCWs may have produced more durable anti-spik antibodies than anti-nucleocapsid. This suggests the existence of a higher SARS-CoV-2 seroprevalence among general population albite somewhat lower than that existing among HCWs although the immune role of the produced antibodies in term of durability and the efficacy remains to be elucidated.

The high proportion of HCW who reported either unacceptance or hesitancy to take the COVI-19 vaccine highlights a concerning misconception that is likely to be prevalent among the general population. This will be a major barrier towards development of efficient COVID-19 vaccination program.

Our study found no significant association (*P* > 0.05) between seroprevalence and gender, age, residence, professional work experience, occupation categories and workplace. This suggests that all HCWs were equally at the same level of risk. These results are consistent with previous studies conducted elsewhere [14, 18, 21]. The absence of significant association between seropositivity and chronic comorbidities and tobacco consumption is in agreement with a study among HCWs elsewhere [43]. The high rate (28.0%) of seropositive HCWs who had never been diagnosed with COVID-19 in the past or those who were asymptomatic (72.0%) infers that a considerable proportion HCWs had subclinical infections and had been carrying out their clinical practice while infected and thus spreading the infection among their patients and among other health personnels. Other findings reported elsewhere suggest a similar scenario [44] which further emphasize the necessity of promotion of IPC measures.

A small number of HCWs with a history of COVID-19 reported undergoing PCR testing to confirm their infection. This points to inadequate adherence to local and international policies regarding testing suspected COVID-19 cases particularly for HCWs, possibly due to fear of stigma, isolation, feel as it was unnecessary, and a shortage of tests supplies, as reported in any other studies [45, 46].

Seroprevalence did not significantly associate with COVID-19-related symptoms including, shortness of breathing, cough, anosmia or loss of taste, and fever/chills. Similar finding have been reported from Saudi Arabia where a sizable proportion of seropositive HCW had not been previously diagnosed with COVID-19 [47].

### Strengths and limitations

The study has the merit of being the first to estimate the seroprevalence of SARS-CoV-2 among HCWs in a minor governorate in Yemen, two years after the start of the pandemic and at the fifth wave. This study showed a significant value as it provides insights into the epidemiological status of COVID-19 and its burden in Yemen.

However, the generalizability of the findings is limited as the study was conducted in only two governorates out of a total 22 in the country. Additionally, the used serological test was only qualitative rather than quantitative, so it does not determine the quantitative value or the rate of the increased levels of antibodies to SARS-CoV-2. Furthermore, there might be several potential sources of bias, including information recall bias on reporting of previous infection with SARS-CoV-2, the use of PPE, and attending IPC training as well as limitation related to the self-completeness of the questionnaire (ignorance of questions, or different interpretations of the question). Finally, the study did not assess antibodies from vaccination, particularly anti-spike, which is an important limitation.

## Conclusions

The study findings highlight the urgent need for improving infection prevention and control measures among HCWs in Yemen, as well as increasing vaccination coverage. The high seroprevalence among HCWs may also suggest a high level of seroprevalence among the general population, emphasizing the importance of continued surveillance and monitoring of the epidemiological situation in Yemen.

SARS-CoV-2 seroprevalence and its impact on the clinical outcomes of infection in term of severity and duration in HCW need to be regularly monitored. Vaccination campaigns should be enhanced according to the WHO current recommendation and assessment of the vaccine. In summary, this study highlights the high burden of SARS-CoV-2 infection among HCWs in Yemen and underscores the need for urgent action to improve infection prevention and control measures and increase vaccination coverage to protect HCWs and prevent the further spread of the virus.

## Data Availability

The data that support the study findings of the current study are available from the corresponding author on reasonable request via this email: bawazir56@gmail.com.
